# Curved display based on programming origami tessellations

**DOI:** 10.1038/s41378-021-00319-1

**Published:** 2021-12-03

**Authors:** Yang Deng, Weixuan Liu, Yik Kin Cheung, Yongkai Li, Wei Hong, Hongyu Yu

**Affiliations:** 1 Department of Mechanical and Aerospace Engineering, Hong Kong University of Science and Technology, Kowloon Hong Kong, SAR 999077 China; 2 Department of Mechanics and Aerospace Engineering, Southern University of Science and Technology (SUSTech), Shenzhen, Guangdong China

**Keywords:** Optical materials and structures, Electrical and electronic engineering

## Abstract

Curved displays have recently become very popular, with wide applications for both industry and consumers. However, built upon initially flat films, most flexible displays are often incompatible with general nondevelopable surfaces. In this paper, we report a method for producing curved displays of nondevelopable shapes by using a structure-mechanics-inspired functional optimization method to design tessellation patterns that fold into the desired shapes. Representative displays in spherical and saddle shapes are demonstrated. The microfabrication process is employed for manufacturing 2D flexible foldable circuit boards, pick-and-place technology is used for placing illuminant elements onto the boards, and mold guidance is used for folding 2-D sheets into curved 3D display prototypes. The proposed technology is feasible for mass production and advances the application of next-generation curved displays.

## Introduction

Curved surfaces, which seem ubiquitous in our daily lives, are desirable for designers for aesthetic and/or practical considerations. Curved electronics^1^ have recently undergone rapid development, covering broad application areas including wearable sensors, smart skins, curved optics, smart appliances, soft robotics, and even home decorations^2–10^. As an indispensable way to access information and gestures, curved displays^11,12^ are one of the key components for curved electronics and constitute functional and attractive next-generation displays.

One way to realize a curved display is by bending a flexible display. Flexible displays^13–15^ have been developed extensively and have mainly been used in household appliances and consumer electronics. As a typical example, organic light-emitting diode (OLED) displays can be fabricated on flexible substrates to achieve flexibility^16,17^ and have already been commercialized. A novel organic liquid crystal display uses an organic thin-film-transistor (TFT) array on a plastic film to replace amorphous silicon TFTs and has achieved a certain amount of flexibility^18^. However, these types of displays are flexible but nonstretchable. According to Gauss’s Theorema Egregium in geometry^19,20^, due to the constancy of the Gauss curvature (GC, K) of a surface under local isometry, flexible but nonstretchable displays cannot be bent into nondevelopable surfaces with nonzero GC; the commercial products mentioned above can only be bent into developable surfaces such as tubes or cones.

Therefore, stretchability is essential for achieving generally curved displays. Stretchability can be realized by mounting rigid devices on a stretchable substrate or by connecting island-type small devices with serpentine interconnects. The application of stretchable materials in curved displays should overcome the difficulties associated with nondevelopable surfaces^1^. Larson et al.^21^ reported an AC-driven stretchable capacitive display. The ZnS phosphors were embedded in Eco-flex 00–30 to emit light when an AC voltage was applied to the two electrodes made by an elastomeric matrix (polyacrylamide, PAM). Tsuyoshi et al.^12^ reported a stretchable active-matrix display that integrates printed elastic conductors (fluorinated rubber), organic transistors, and OLEDs to sustain 30–50% strain and that can be wrapped on a spherical surface. These stretchable displays can fit different nondevelopable surfaces by stretching. Unfortunately, stretchable materials such as polydimethylsiloxane (PDMS), Eco-flex rubbers, and poly(3,4-ethylene dioxythiophene) (PEDOT)^22^ often suffer from low stiffness and short fatigue life, resulting in the luminescent or conductive materials embedded in them being damaged due to large deformation. Another strategy for stretchability is to employ geometrically stretchable interconnects to connect rigid islands of illuminant elements. Rogers et al. developed a technology that used serpentine-shaped interlinks to connect pixels, and the connected μ-ILED array was then transferred to a 400 μm-thick prestretched PDMS sheet. The serpentine microribbons detached from the substrate and formed noncoplanar pop-up bridges after releasing the prestrain; the array reached 48% stretchability and was compatible with spherical surfaces^23^. Tripathi et al.^24^ also reported a 32 × 32 active matrix light-emitting diode (AMLED) stretchable display in which the pixels are connected via 2 mm long horseshoe metal interconnects. Materials for this type of stretchable display still need to work as holding substrates, which results in limitations similar to those of previous types of devices. Furthermore, nearly all curved displays rely on stretchable interconnects and encounter problems including limited deformability, complex manufacturing processes, high costs, and low yields that hinder mass production.

Origami-based structures, as demonstrated in this paper, can outperform these previous structures. Origami^25,26^, an ancient form of papercraft, is the folding of 2D sheets into 3D structures through specifically designed patterns. The stress and strain caused by folding are highly localized at the creases and suppressed on the facets. The rigid foldability of origami-based structures enables compatibility with rigid components by placing them on the facets. Miura-ori^27–29^, a widely recognized form of origami with desirable properties (such as developability and rigid foldability), has been used in stretchable devices such as circuit boards, photodetectors, batteries, solar cells and lighting devices^30–33^. In origami design, Dudte et al. demonstrated mathematically that surfaces of given curvatures can be achieved by folding origami structures with optimized tessellations via elementary geometric constructions^34^. However, the rigid foldability requirement has to be relaxed by adding a diagonal crease to each facet, which is incompatible with typical rigid components. In addition, the inhomogeneity in the facet size in the optimized design may cause difficulties in manufacturing.

In this study, by taking the bending stiffness of facets into consideration, we develop a different approach through the design of Miura-like origami tessellations that can be folded into nondevelopable surfaces with minimal in-facet bending and that remain compatible with existing high-throughput manufacturing processes. The optimization algorithm minimizes the pseudostrain energy of all facets, thus minimizing the deformation on the facets of origami during folding. A special finite element scheme is used to calculate the weak-form functional extremum in classical differential geometry and helps programmatically design the specific 2D origami pattern. The silicon-based microfabrication process, pick-and-place packaging process, and mold-based origami folding process are integrated for manufacturing curved LED displays of nondevelopable surfaces, such as spherical and saddle surfaces.

## Results and discussion

### The design principles of flexible and foldable displays

**Fig. 1:**
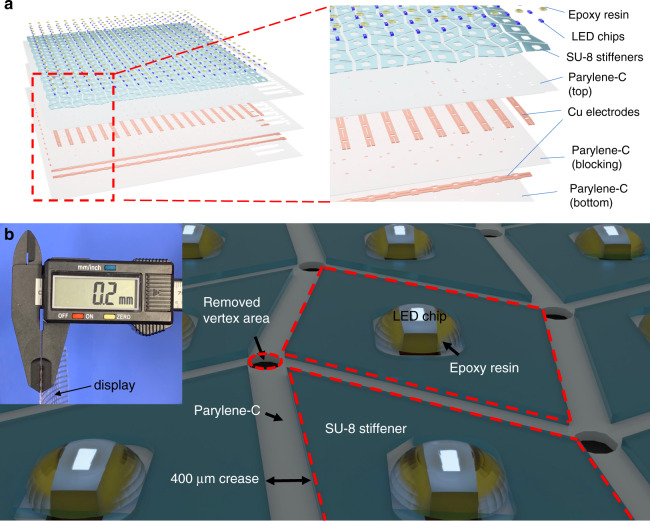
The structure and rendering of the display. **a** Schematic diagram for the multilayer structure of the origami-enabled curved display prior to folding. The materials and morphology of each layer are identified in the partially magnified view. **b** 3D rendering of the display. Yttrium aluminum garnet (YAG) and resins are used to encapsulate LED chips, and the inset shows the total thickness of the display

A foldable 2D LED display sheet is manufactured first. The multiple-layer structure of the display is illustrated in Fig. 1. Fig. 1a shows a flexible substrate consisting of three layers of parylene-C (10 µm bottom layer, 5 µm blocking layer, and 5 µm top layer) and two layers of 0.5 µm Cu as electrodes, including row and column address lines. Reactive ion etching (RIE) is used on the parylene-C to expose the copper electrodes for bonding with LED chips and to remove the vertex areas (the red dashed circle in Fig. 1b) between the SU-8 quads (detailed in the discussion section) to reduce the stiffness. A 100 µm-thick SU-8 stiffener covers the major part of a facet (marked with the red dashed boxes), with a rounded square hole punched in the center to house the LED chip. The SU-8 stiffeners are smaller than the facets, leaving a 400 µm-wide space near folding creases to ensure safe bending clearance at the creases. The sharp corners of the SU-8, as shown in the schematic diagram, are naturally rounded in manufacturing. LED chips (approximately 150 µm thick) are picked up, placed, and soldered in the reserved rectangular area at the center of every SU-8 stiffener and then encapsulated with dielectric epoxy resin. The total thickness of the assembled 2D foldable display is approximately 200 µm, while the thickness at the crease regions is only 20–30 µm.

### Unfolded display in the planar configuration

**Fig. 2:**
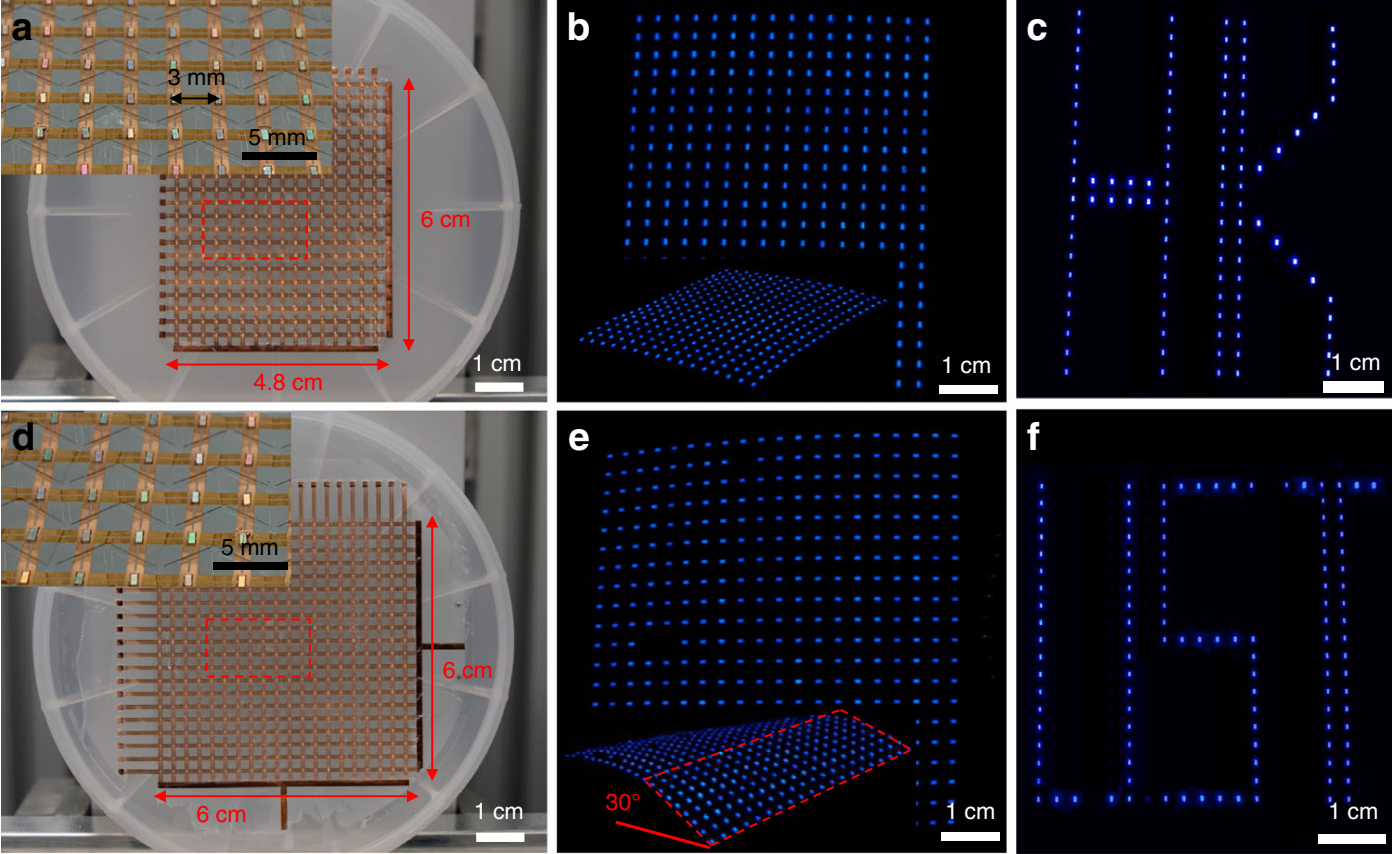
Images of flat displays. **a**–**d** Photographs of unfolded spherical and saddle-shaped displays. The insets are magnified views of the regions in the red dashed boxes. **b**–**e** Top views of lit-up displays. Insets show the tilted views of the flat configuration. **c**, **f** Photographs of the displays presenting letters

According to the origami patterns specifically optimized with the algorithm detailed in the Methods and fabrication section, planar sheets for spherical and saddle-shaped displays are fabricated and demonstrated before folding. Fig. 2 presents optical images of the devices. A 4.8 cm × 6 cm spherical display with a 16 × 20 LED array and a 6 cm × 6 cm saddle-shaped display with a 20 × 20 LED array are shown in Fig. 2a and d, respectively. The magnified view of the red dashed boxes shows the LED chips with a 3 mm pixel pitch. The LED chips are individually electrically driven for display purposes with a passive matrix configuration. Fig. 2b and e demonstrate the powering up of all the LED pixels on the two displays. A few pixels are damaged during the LED soldering process, and the yield is higher than 95%. The passive line scan control system provides a signal pulse frequency of 100 Hz (detailed in the Supplementary Materials). The insets of Fig. 2b and e show diagonal views of the flexible and foldable display sheets. Due to the small thickness and the flexibility, the suspended part (highlighted via the red dashed frame in Fig. 2e) bends downward and forms a 30° dihedral angle with the horizontal plane, which means that it is naturally compatible with a developable surface. Supplementary Movie 1 demonstrates that the 2D display sheet can work as an excellent flexible display. The video shows that the display works steadily as it undulates like a wave, which suggests its excellent dynamic stability. As shown in Fig. 2c and f, the letters “HK” and “UST” are presented by the two displays (video clips available in Supplementary Movie 2).

### Folded curved displays

**Fig. 3:**
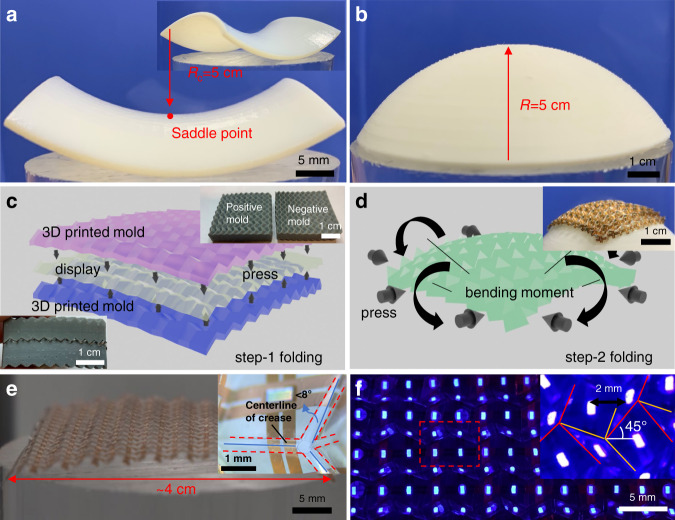
The folding process and morphological characterization of the folded display. **a** Front and angled views of a 3D-printed mold for hyperbolic paraboloids, with a 5 cm radius of curvature at the saddle point highlighted. **b** 3D-printed hemispherical mold with a 5 cm radius. **c** Schematic diagram showing the display sandwiched between a pair of partially folded curved molds. Mountain and valley creases are formed row by row by applying pressure to the molds. **d** Lateral squeezing forces and bending moments are simultaneously applied to the device to deform it toward the preset topography and fixed on the corresponding curved surface (inset). **e** Optical image of a folded display before bending. Its length is shortened to approximately 4 cm after step-1 folding. The inset shows a schematic diagram of the misalignment of the centerline of the creases. **f** LEDs lit-up in the folded configuration and the magnified view showing the mountain and valley creases and 2 mm pixel pitch

Following the programmed spherical and saddle-oriented origami pattern designs, a saddle surface mold with a 5 cm radius of curvature at the saddle point (K = –0.048 cm^−2^) and a spherical mold with a 5 cm radius (K = 0.04 cm^−2^) are fabricated via 3D printing (the analytic expressions of surfaces are presented in the Methods and fabrication section). The front view (Fig. 3a) and the angled view (inset) of the saddle surface illustrates the principal curvatures. With the aid of 3D molds, the displays are folded from flat 2D configurations into 3D stretchable structures. When compressed, a typical Miura-Ori structure can exhibit either the origami-dominated mode or a global-buckling mode, depending on structural parameters and constraints^31^. An energy-based optimization algorithm and SU-8 stiffener-based modification (detailed in the following section) ensure the domination of the origami mode. Fig. 3c, d demonstrate the stage-1 and stage-2 origami folding processes. Generally, there are two steps to fold a 2D display into 3D geometry. The first step is to inscribe the mountain and valley creases by pressing the flat display with 3D printed folding molds (insets of Fig. 3c). The folding molds (a pair of positive and negative molds that match each other) consist of optimized origami patterns in the state before bending into a corresponding curved surface. The pixel at one corner is utilized as the alignment mark for the folding process, and the 2D display adaptively shrinks and matches the mold when gradually sandwiched in the molds. After the mountain and valley creases are inscribed, the display is further bent and fixed on the corresponding curved surface (Fig. 3d and inset). Fig. 3e shows a folded display with a folding angle of approximately 45° from flat for each facet or ∼30% nominal biaxial strain from the initial flat configuration (the stretchability and morphology of the folded display are analyzed in the Supplementary Materials). As the folding process is dynamic, there are always misalignments between the molds and creases. The flexibility of 3D-printed modes and the designated gaps between SU-8 stiffeners accommodate the misalignments and establish the robustness of the folding process. The accuracy of alignment after folding is measured under an optical microscope. This illustrates that the maximum torsion angle of the crease centerline is 8°, and the offset at both ends of the crease is 200 µm in this case (inset of Fig. 3e). The pixel pitch is reduced to approximately 2 mm, as shown in Fig. 3f. The insets are magnified views of the lit-up LED chips, in which the mountain and valley creases are marked with red and yellow lines, respectively. Importantly, the optimization algorithm detailed as follows manages to maintain an approximately constant center-to-center distance between neighboring facets for easy mounting of LED chips and a better display effect.

**Fig. 4:**
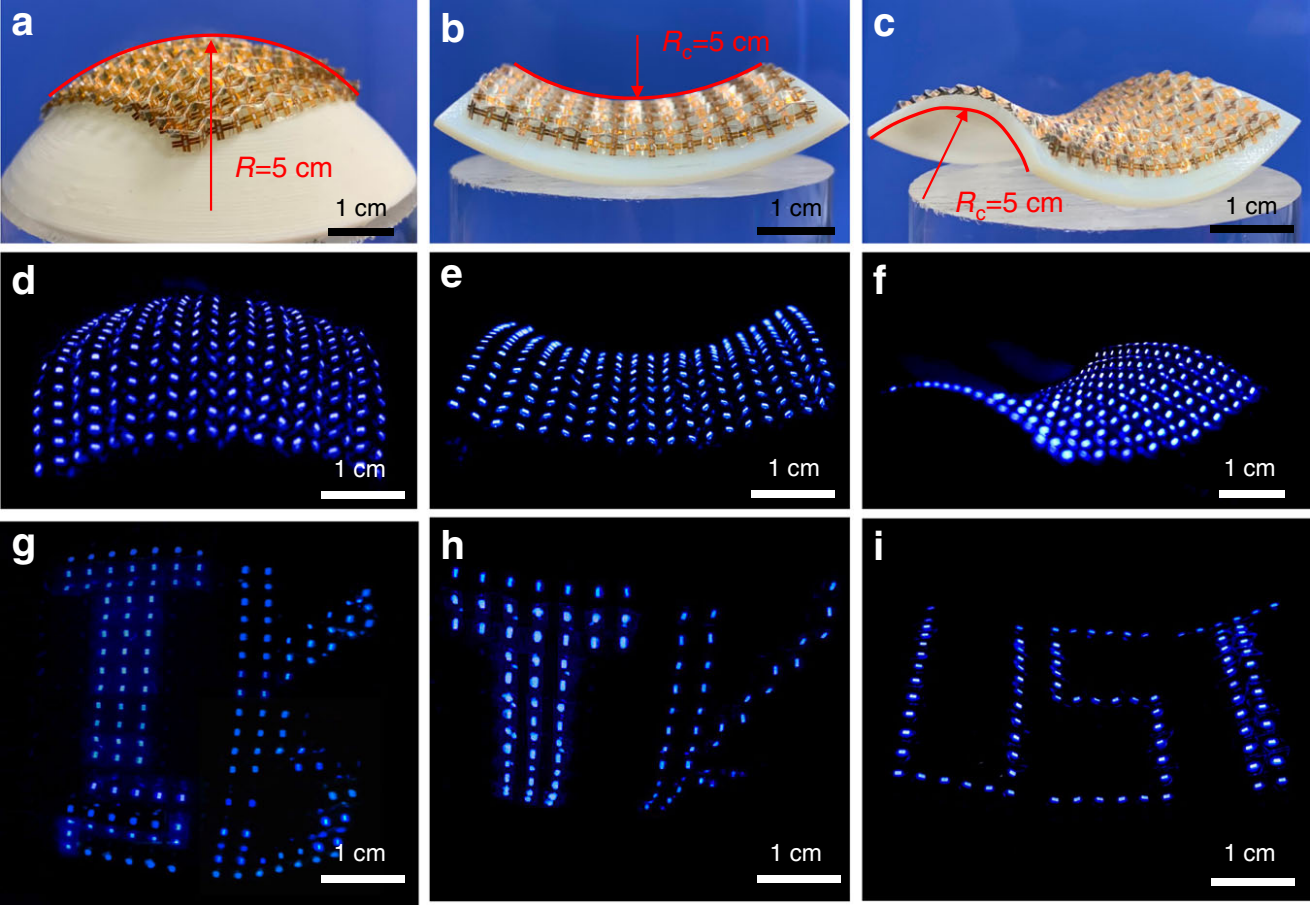
Images of curved displays. **a** Photo of the spherical display fitted on a spherical mold. **b**, **c** Front and diagonal views of the saddle display. The red curves indicate the parabolas forming the saddle-shaped surface, one opening upwards and the other opening downwards. **d** Front view of the lit-up spherical display. **e**, **f** Saddle-shape display with all LEDs lit. **g**, **h** Top and front views of the spherical display presenting the letter pattern “TK2U” (“2U” not seen in **h** due to the angle of view and is detailed in Supplementary Materials for the saddle-shaped display). **i** Angled views of the saddle-shaped display showing capital letters “UST”

Curved displays are correspondingly placed on spherical and saddle surfaces. Fig. 4a shows the angled view of the spherical display fitted on a hemisphere of radius 5 cm. Fig. 4b, c show the front and angled views of the saddle display, respectively. The two parabolas (highlighted by red curves) forming the hyperbolic paraboloid have the same curvature but opposite directions at the saddle point. Fig. 4d–f presents the states of the two curved displays when all LEDs are lit-up. Photos of the displays in operation (showing letter patterns) from different angles of view are given in Fig. 4g–i (video clips available in Supplementary Movies 3 and 4). The image of the display in the curved configuration is slightly distorted.

### Tessellation design and modification

Theoretically, distortion occurs on each parallelogram if the folded Miura-ori structure is forcibly mounted on a curved surface, and strain energy is generated accordingly. In our optimization method, we grant the vertices of the Miura-ori structure extra degrees of freedom. Following the “minimum total potential energy principle”, vertices can adjust to the best position to achieve the lowest strain energy of the whole system. In this way, for the optimized origami structure, the maximum strain of the system is effectively reduced.

**Fig. 5:**
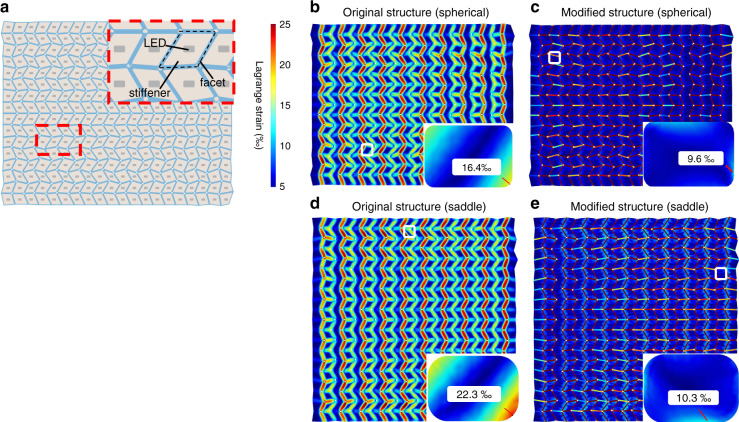
Geometrical model and strain analysis of optimized patterns. **a** Geometrical model of the optimized origami pattern for a spherical display. **b**, **c** Principal strain distribution of original and optimized spherical origami patterns. The insets show the central regions of the SU-8 facets (marked with the white boxes) with the maximum strain in the corresponding pattern. **d**, **e** Principal strain distribution of original and optimized saddle-shaped origami patterns. Similarly, the insets indicate the maximum strain in each pattern

In reality, the material at the creases produces a nonnegligible strain and spreads to the LED region of facets. The difference between the bending stiffnesses of facets and creases can significantly reduce the strain in the LED region. To further reduce the maximum strain in the LED region, SU-8 stiffeners are introduced to increase the bending stiffness of the facets, and holes are drilled in the vertices to reduce the bending stiffness of the creases. Both modifications can ensure the domination of the origami mode instead of the global-buckling mode^31^. According to the actual parameters, COMSOL 5.4 is used to establish the geometric model of the display (Fig. 5a). A solid mechanics physical field and a time-dependent analysis step are used for simulation. The initial structure is a flat surface and is divided into shell elements. The real Young’s moduli and Poisson’s ratios of SU-8 and parylene-C are used in linear elastic materials. A weak contribution is used to construct a penalty for the facets to compel the specified displacement to match the corresponding curved surface. Fig. 5 shows the resulting strain distributions in various cases. As shown in Fig. 5b and d, prior to modification, when the Miura-ori structure is bent into spherical and saddle shapes, the strain generated spreads onto the facets, which can easily lead to debonding of the LED chip from the substrate. For the optimized origami pattern, the strain is highly localized in the crease regions when bent to the same shape, as shown by Fig. 5c and e. As shown by the insets of Fig. 5b–e, the maximum strain in the central regions of the SU-8 facets (the reserved rectangle area for the LED chip) is reduced by ~41% (from 1.64% to 0.96%) for the spherical shape and ~54% (from 2.23% to 1.03%) for the saddle shape. The greatly reduced strain on the facets ensures mechanical and electrical reliability. In addition, the relevance between the strain and shapes and sizes of the origami structure is discussed in the Supplementary Materials.

## Discussion

**Fig. 6:**
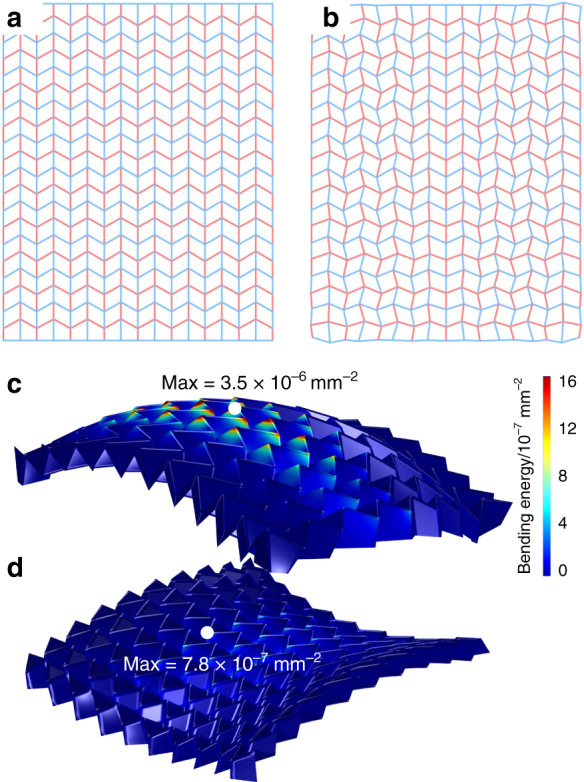
The optimization process for typical Miura-ori to optimized origami pattern. **a** Typical Miura-ori pattern consisting of parallelogram tessellations. Mountain and valley creases are presented with red and blue lines, the base and height of the parallelogram are both 3 mm, and the acute internal angle α = 60°. **b** The optimized origami patterns for a spherical shape. **c**, **d** Modified origami patterns are folded to approach given spherical and saddle surfaces (the maximum bending energy is given)

Compared with Dudte’s work, our optimization method has several advantages. First, previous work hypothesizes that the diagonal edge of the facet can also be folded by attaching a bending stiffness to it, allowing the origami structure to cover the curved surface. Such deformation would be unacceptable for hard electronic components even when they are small. Our method allows the facet to bend and twist as a plate and realizes a pattern with low bending energy, as shown in Fig. 6 (detailed in the Methods and fabrication section), and a satisfying strain level, as shown in Fig. 5, that is suitable for electronics fabrication.

Second, Dudte’s method is a triangle origami tessellation scheme. Mathematically, triangle origami tessellation can easily cover any surface. Combining triangles two by two into quadrilaterals seems a little tricky and impractical. Our method is a genuine quadrilateral origami tessellation scheme, which is more in line with actual industrial demand.

Third, our method allows us to easily add manufacturing constraints. The pattern designed by Dudte shows a large geometric deviation from standard origami. Additionally, the inner corners of some facets are very sharp in Dudte’s design, which limits engineering applications. Such disadvantages can be easily bypassed by adding the corresponding pseudoenergy in our method.

## Conclusion

Origami folding has been used in electronics for its system-level stretchability. In this paper, we demonstrated the design and fabrication of curved displays by optimizing 2D origami patterns for target 3D shapes. An optimization algorithm minimizing the bending energy in the facets by using a special formulation of the finite element method was employed to design 2D origami tessellations for the desired surface topography. We applied the optimized tessellation patterns to manufacture origami-based curved displays for typical nondevelopable surfaces (spherical and hyperbolic paraboloids). To further reduce stress concentration and enhance reliability, SU-8 stiffeners were attached to the facets, and vertices were removed. Techniques including MEMS-based microfabrication, pick-and-place packaging, and origami folding processes were integrated to fabricate the prototypes. The excellent performance of the folded curved displays illustrate the feasibility of the concept.

## Methods and fabrication

### Optimization of origami tessellations

The tessellation design of an origami structure is formulated into an optimization problem of the map **X**(**X**_0_):**R**^2^→**R**^2^ (detailed background and mathematical modeling can be found in the Supplementary Materials), which identifies the optimal location **X** (Fig. 6b) for each vertex originally located at **X**_0_ in a uniform Miura-ori manner (Fig. 6a). As a general curved surface (especially one with positive Gauss curvature^34^) may not be rigidly folded from a Miura-like tessellation, we turn to the minimization of the total elastic energy of all facets when forced to deform into the desired shape.

The deformation of the tessellated origami pattern is described by another map, **x**(**X**):**R**^2^→**R**^3^, which maps each particle on the 2D plane **X** to the location **x** in a 3D folded state (dynamic deformation processes are available in Supplementary Movies 5 and 6). With the contributions from both out-of-plane bending and considering in-plane stretching, the total elastic energy takes the form

1 $$\begin{array}{*{20}{c}} {{\Pi}\left[ {\boldsymbol{x}\left( \boldsymbol{X} \right)} \right] = {\displaystyle\int}_A {\left[ {\xi \left( {C_{\alpha \alpha } + \frac{1}{{\det \boldsymbol{C}}} - 3} \right) + {\rm K}_{\alpha \beta }{\rm K}_{\alpha \beta }} \right]dA} } \end{array}$$

where **C** is the metric tensor, **Κ** is the Riemann curvature tensor, and repeated indices in Greek imply a summation over the in-plane dimensions. The integral is carried out over the entire 2D plane of the tessellation. We model the facets as linear elastic plates, such that the energy densities of membrane stretching and bending take the form of an integrand, with ξ being an adjustable parameter for tuning the relative importance of the two. As only energy is used in tessellation design, the stiffness parameters of the material are omitted. The total elastic energy Π is used as the objective function and is evaluated through the finite element method for each map **x**(**X**), subject to proper displacement boundary conditions to bend the origami into the desired shape.

It is noteworthy that an ideal origami structure has negligible bending stiffness at the creases relative to the facets. While directly prescribing a small stiffness at the narrow crease regions would be difficult, we intentionally use Lagrange interpolation to approximate the map **x**(**X**). As the fourth-order governing equations of linear plate theory require C1 continuous interpolation in the standard finite element method, C0 Lagrange elements are never chosen. Here, the discontinuity in the slope between neighboring Lagrange elements is employed to model the low bending stiffness at the creases. Because the static equilibrium problem is also a minimization problem, the mathematical problem of tessellation is a composite optimization of the total elastic energy

2 $$\begin{array}{*{20}{c}} {\mathop {{\min }}\limits_{\boldsymbol{X}\left( {\boldsymbol{X}_0} \right)} \left\{ {\mathop {{\min }}\limits_{\boldsymbol{x}\left( \boldsymbol{X} \right)} {\Pi}\left[ {\boldsymbol{x}\left( {\boldsymbol{X}\left( {\boldsymbol{X}_0} \right)} \right)} \right]} \right\}} \end{array}$$

Although the tessellation design only requires the vertex-to-vertex discrete map, we extend the map **X**(**X**_0_) into a continuous map through linear Lagrange interpolation to facilitate numerical calculations.

While the abovementioned approach already yields satisfying results if just the origami design is of concern, the actual manufacture and applications pose additional constraints. In an LED display, for example, the pixels are usually placed in a rectilinear array to avoid distortion. Additionally, a uniform facet size is preferred to accommodate the on-facet components. Obviously, these constraints can never be rigorously satisfied in the general case. Here, we try to address them through a penalty method – by the addition of additional positive definite terms in the energy functional Π. To better align the on-facet components, we add the term

3 $$\begin{array}{*{20}{c}} {\eta _1\mathop {\displaystyle\sum}\limits_e {\mathrm{abs}\left( {\bar x_\xi - \bar x_\xi ^0} \right)} } \end{array}$$

to bring the center of each quadrilateral facet (element), ${{{\bar{\mathrm X}}}}$, closer to the corresponding facet center ${{{\bar{\mathrm X}}}}^{{{\mathrm{0}}}}$ in a standard Miura-ori design. The latter is calculated from linearly scaling the corresponding vertex coordinates **X**_0_. Term (3) is summed over all elements, and η_1_ is a positive tuning parameter. Similarly, the uniformity in facet size is enforced by adding the term

4 $$\begin{array}{*{20}{c}} {\eta _{2}\displaystyle\mathop {\sum}\limits_{e} {\left[ {{\displaystyle\int}_{\!\!e} \det \frac{{\partial \boldsymbol{X}}}{{\partial \boldsymbol{X}_{0}}}dA_{e} - A_{e}} \right]^{2}} } \end{array}$$

in which the integral is carried out over the area of each facet element A_e_ and η_2_ is another tuning parameter.

The minimization problem (2) is written into a variational form, discretized into quadrilateral elements, and implemented in the commercial finite element platform COMSOL Multiphysics 5.4, as detailed in the Supplementary Materials. The typically optimized tessellations, which fold into spherical and saddle-shaped origamis, are shown in Fig. 6, together with the folded geometries. The bending energy plotted in Fig. 6c and d is calculated by Κ_αβ_ Κ_αβ_. The maximum bending energy of each tessellation design is much lower than the equivalent bending energy of the corresponding curve surface, which is 8 × 10^−4^ mm^−2^ for the original spherical surface and 9.7 × 10^−4^ mm^−2^ for the saddle surface, allowing the hard component to attach. Additionally, the bending energy of the saddle-shaped design is much lower than that of the spherical design, which conforms to Dudte’s theory stating that rigidly foldable Miura-like tessellations exist on negative Gauss curvature surfaces but not on positive ones.

### Fabrication

**Fig. 7:**
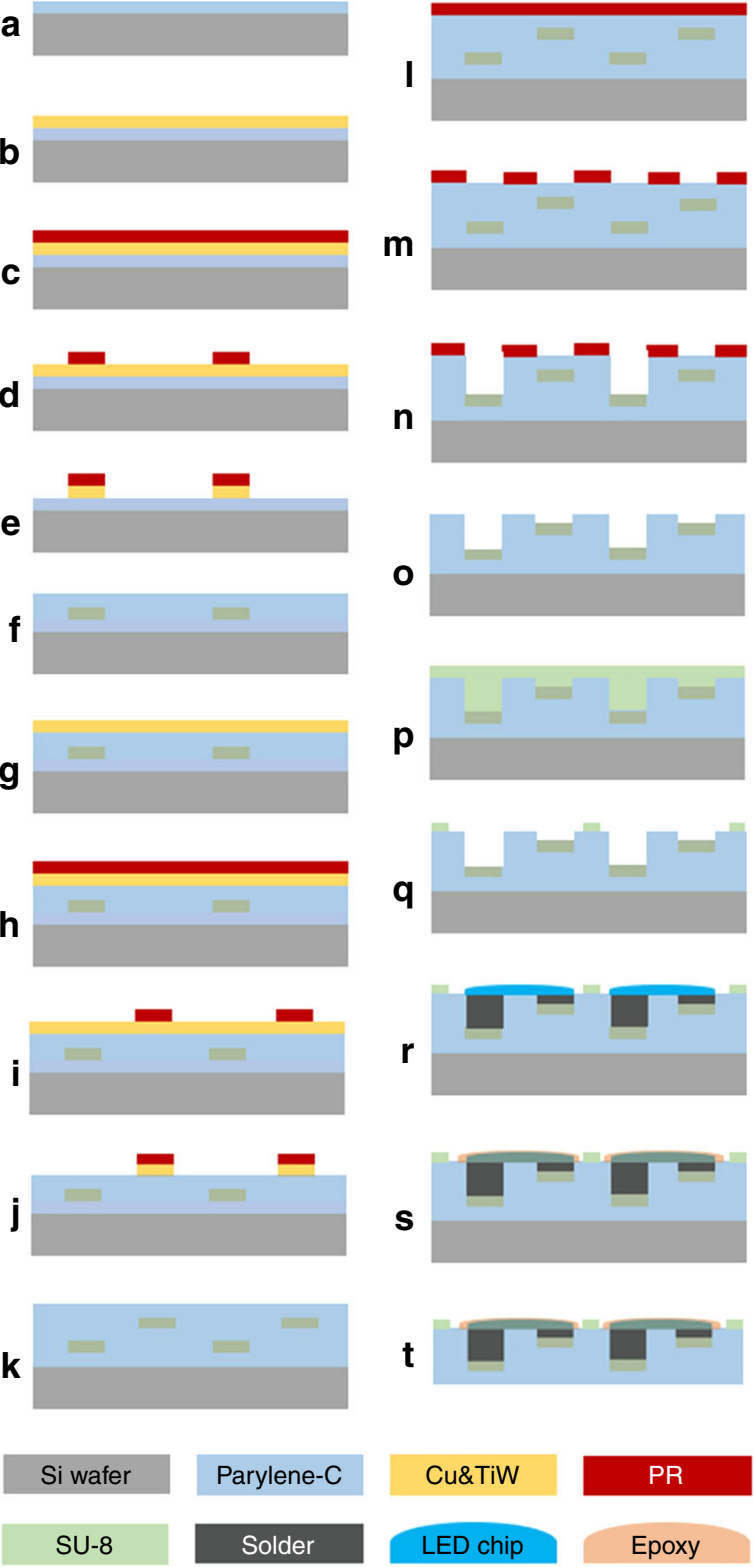
The MEMS-based fabrication process flow for a flexible circuit film. **a**–**e** The processes for patterning the first layer of circuit. **f** The blocking layer of Parylene-C deposition. **g**–**j** The processes for patterning the second layer of circuit. **k** The top layer of Parylene-C deposition. **l**–**o** Opening the contact pads on the flexible circuit. **p**–**q** The processes for patterning SU-8 stiffener. **r**–**t** LED chips packaging and encapsulation

MEMS-based foldable circuit substrate fabrication and LED pick-and-place packaging processes are detailed as follows (Fig. 7).

First, the bottom parylene-C (Galentis) layer was deposited with an SCS Labcoter 2 (PDS 2010) vacuum deposition system. The silicon wafers were placed on the quartz boat at a certain distance during the process to obtain a uniform thickness film and to protect its surface from being scratched. Then, bottom electrode sputtering deposition was performed. With a high sputtering rate (~175 Ȧ/min for TiW and ~1000 Ȧ/min for Cu), approximately 300 Ȧ titanium-tungsten (TiW, works as an adhesion enhancer between the Cu and parylene-C film) and 5000 Ȧ Cu were sequentially sputtered on the bottom parylene-C film using a vacuum sputter deposition system (CVC-601).

The adhesion promoter hexamethyldisilazane (HMDS) was deposited on the metal layer in a negative pressure chamber before photoresist (PR) coating. AZ 504 as a positive tone PR and a SUSS Coater (PHT-SC1) spin coater were selected for patterning Cu electrodes. The AZ 504 reached approximately 1.1−1.2 µm when two-step coating was performed with 500 rpm for 5 s as step 1 and 4000 rpm for 30 s as step 2. A soft bake process (110 °C for 60 s) on a hot plate was performed next to drive off the solvent and to solidify the film. Then, exposure with an AB-M Aligner (UV with power 22 mW/cm^2^) for 5–5.5 s and developing using the positive resist developer FHD-5 (the concentration of tetramethylammonium hydroxide (TMAH) was 2.3%) for 60–70 s were performed. After the residual developer was washed away with deionized water, the surface of the samples was dried with a nitrogen gun. To ensure complete removal of solvent and to improve adhesion in the following wet etching process, a hard bake at 120 °C for 30 mins in an oven was needed.

Asher (IPC 3000 with 100 Ȧ/min PR etching rate) was selected for the descum process to remove the residual PR scum. After that, the samples were immersed in a mixed solution of dilute sulfuric acid and hydrogen peroxide (H_2_O: H_2_SO_4_: H_2_O_2_ = 40: 10: 1) to etch the Cu. Then, they were placed in hydrogen peroxide at 60–70 °C to etch the TiW.

The deposition of the blocking layer of parylene-C, the patterning of the second layer of Cu electrodes, and the deposition of the top layer of parylene-C followed sequentially. After that, AZ 9260 was selected as the masking layer for opening the contact pads and removing the vertex areas of parylene-C. AZ 9260 reached 16–17 µm under a two-step coating process with 500 rpm for 5 s as step 1 and 1000 rpm for 30 s as step 2. Correspondingly, the soft bake time was extended to 250 s at 110 °C on the hotplate. Then, the AB-M aligner was used for a 70 s exposure, and the FHD-5 was used for 5 mins of developing. The RIE system (at an etch rate of 0.5 µm/min) was used for stripping parylene-C, and the positive resist stripper MS-2001 was used to remove the residual AZ 9260. A desktop coater (PHT-SC2) was used to spin coat SU-8 2075, and the soft bake process on the hotplate (5 mins at 65 °C and 10–20 mins at 95 °C) was performed next. The SU-8 film was exposed for 13 s, and a postexposure baking (PEB) step was needed to provide energy for the continuous reaction of the included photoactive component. After developing with SU-8 developer for 7–10 mins, the wafers were hard baked at 150–250 °C for 5–30 mins in the oven to stabilize their properties.

A die bonder (ASM AD860) and reflow oven (Sun east IPC780E) were used for the LED pick-and-place packaging. Considering the thermal properties of parylene-C, SRA low-temperature lead-free solder paste (42Sn/57Bi/1Ag) was selected to bond LED chips (15 mil × 30 mil Bridgelux blue power die) with the flexible circuit. After that, to prevent detachment between the LED chips and flexible substrate, an automatic dispenser (Camalot 1414) and epoxy resin were needed to encapsulate the chips. After the epoxy resin was cured, the display was peeled off from the Si wafer.

## Supplementary information

Flexible display

Flat displays presenting letters

Saddle-shaped display presenting letters-1

Saddle-shaped display presenting letters-2

Saddle-shaped origami tessellations

Spherical-shaped origami tessellations

Supplemental materials
