# Supplementary material for: Curved display based on programming origami tessellations
Source: Microsyst Nanoeng. 2021 Dec 3;7:101. doi: 10.1038/s41378-021-00319-1 (PMC8642556; doi:10.1038/s41378-021-00319-1)
Supplement: Supplementary file 7 — Supplemental materials [file 41378_2021_319_MOESM7_ESM.docx]

**Supplementary materials**

**Driver circuit design**

The LEDs are driven by a shift register (74HC595) circuit which is simple to design and layout. Also, the shift register can cascade by feeding the final bit of data into the next shift register. Therefore, an 8x8 LED matrix driver can be expanded to 16x16 and 32x32 by cascading.

The schematic of the driver circuit is shown in Figure S1. We only show the driver for 1 row inside an 8x8 LED matrix. There is a 500$\Omega$ resistor series connected to the output pin of the column selector to ensure the output current is within the total load current (70mA) for 8 simultaneous to light-up. In operation, there are 6 inputs from the Arduino controller which are 2 data pins (DS), 4 pins (STCP, SHCP) to control shifting and refreshing operations of column selectors and row selectors, respectively. The corresponding LED is active when the output of the row selector is set to low, and the output of the column selector is set to high. To individually control the LEDs, the Arduino program will scan through the LED matrix row by row, and the output of the column selector changes according to the desired light-up pattern for each row.

**Fig. S1 a** Schematic diagram of drive circuit system for 8x8 LED matrix. **b** The pin assignment of shift register 74HC595 and logic diagram of the circuit.

**Stretchable performance and morphology characterization**

Certainly, our curved display does have a certain degree of stretchability. The configurations of the display before and after folding is demonstrated in the article (Fig. S2 a, b). Moreover, the display can be pressed further till the folding angle on the creases is reduced by 20° and the overall area reduce by 55% with reliable function. The linear stretchability along X axis is $\varepsilon^{x}=\frac{X_{\max}-X_{\min}}{X_{\min}}=33.3\%$ and Y axis is $\varepsilon^{y}=\frac{Y_{\max}-Y_{\min}}{Y_{\min}}=66.6\%$ (Fig. C5 c). The [Poisson's ratio](javascript:;) in XY plane is calculated as $\nu=-2$.


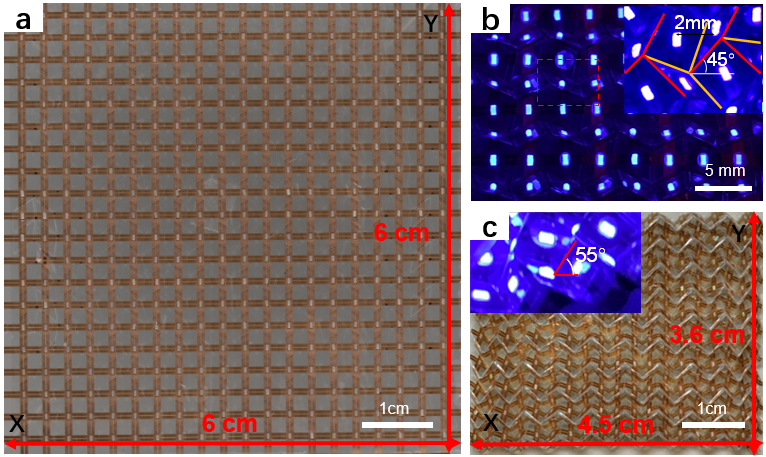


**Fig. S2** Optical images showing the stretchability of the device. **a** The display in flat configuration. **b** The mountain and valley creases of the display are inscribed via folding molds. **c** The morphology of folded and pressed display.

To understand the structure of our curved display comprehensively, it is necessary to analyze morphology and statics more. Fig. S3 a shows the optical image of 2x2 pixels and all the marks, morphology features and sizes correspond to the 3D rendering in Figure 1 in the article. Fig. S3 b shows the morphology of the display in a fully folded configuration taken from the back. It is comprehensible that α is the dihedral angle between facet 1 and the facet 2 near the brown creases (brown dotted line Fig. S3 b). In the multilayer substrate, the distance between the copper electrode layer and the neutral surface is estimated as 5 µm. According to the general formula for bending normal strain, the strain at this layer is estimated to be about 1.3%. Even for the crease with smallest folding angle, the strain at copper layer is estimated to be about 3.4% which is far less than the elongation at break (20%) of the material. It can be seen from Fig. S3 c (and partial enlarged Fig. S3 d) that the wrinkles caused by excessive plastic deformation did not appear ^[31]^.


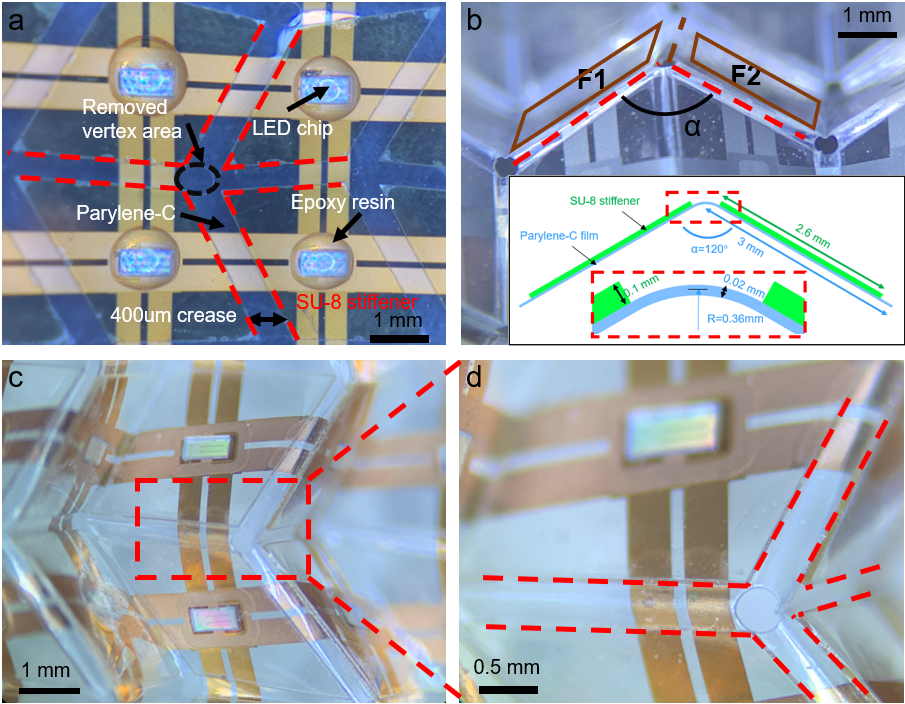


**Fig. S3** **a** Optical image of display in flat configuration. **b** Optical picture of fully folded display taken from the back and its geometric schematic diagram in this configuration (inset image). **c** Optical picture of fully folded display taken from the front and its partial enlarged view **d**.

[Besides](javascript:;), the ratio of the quads’ diagonal length of the optimized origami pattern was analyzed to characterize the shape of facets. As shown in Fig. S4 a, b, the smallest ratio of the quads’ diagonal length of spherical and saddle origami pattern are 34% and 30%, respectively. Compared with the original parallelogram tessellations (58%), some quads are longer and narrower. Even so, the optimized patterns found a balance between meeting the requirements of display pixel pitch, yield and space to accommodate the LED chip, to achieve the desired effect.


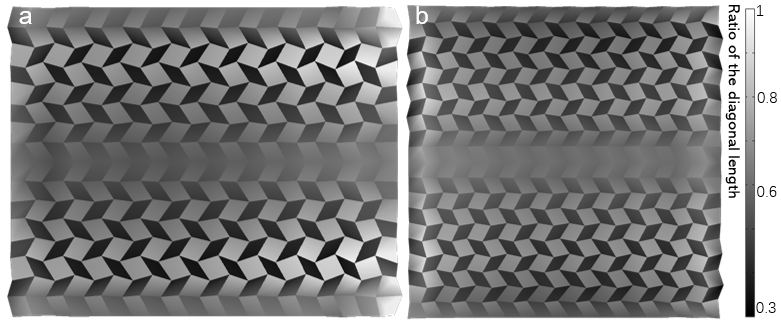


**Fig. S4** The ratio distribution of the quads’ diagonal length of the optimized origami patterns.

**Visual range analysis**

Compared with spherical display, the visual range of saddle display is more complicated. As mentioned in the article, the saddle surface is composed of two parabolas with opposite curvatures. Thus, the visual range perpendicular to each parabola is different. As shown in Fig. S5a, c, e, the viewing angle is perpendicular to the parabola with positive curvature (highlighted via blue solid line in Fig. S5a), the image “HK” is still recognizable when the angle with the horizontal plane α = 30° and α = 10°. Thus, the visual range is probably 10°＜α＜170°. Similarly, when the viewing angle β is perpendicular to the parabola with negative curvature (highlighted via blue solid line in Fig. S2b), the image is no longer recognized as β = 10° (Fig. S5b, d, f). In this case, the visual range is 30°＜β＜150°.

**Fig. S5 a b** Schematic diagrams present two different directions to observe the saddle-shaped display. **c d e f** Optical images show the visual range (α and β) from the two directions following **a b**.

When the display is curved, the viewing angle of the audience watching the display changes, which is the primary cause for the fluctuation of the display luminance. Here, we estimate the luminance of the overall device by testing the luminance of the LED chip from different tilt angles. Fig. S6 shows that the LED chip is driven by 60mW DC power and tested from different tilt angles. When the dihedral angle between the luminance meter and the LED surface reaches 30°, the luminance reaches 145 cd/m^2^, which is greater than 90% of the highest luminance 160 cd/m^2^. Generally, even when the display is bent, the angle between most of the LED chip and the tangent plane of the curved surface will not be greater than 70°. Therefore, the overall luminance of the display fluctuates less than 10%.


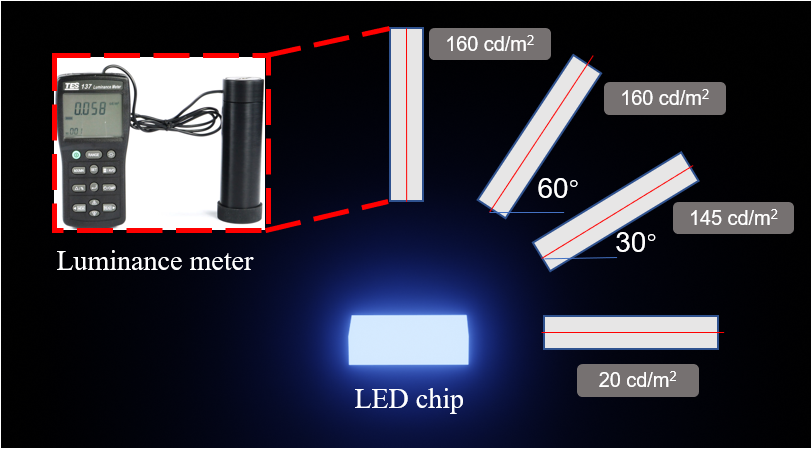


**Fig. S6** Schematic diagram of testing LED luminance from different tilt angles.

**Analysis of the influencing factors of strain**

Generally, the strain mainly depends on the size of the folding angle (the dihedral angle of two adjacent facets), the morphology of the origami facets, and whether there are rigid stiffeners or not. Theoretically, origami facets endure little strain as the strain on creases doesn’t spread out (determined by $\mathbf{K}$ and $\mathbf{C}$ in formula (1)). Fig. S7 a, b shows the maximum principal strain distribution of origami patterns in this situation. In reality, the bending stiffness at the creases cannot be completely eliminated so that causes the strain on facets. The strain on a facet is mainly related to the folding angle of the adjacent creases. The smaller the angle between facets after folding, the larger the in-plane strain.


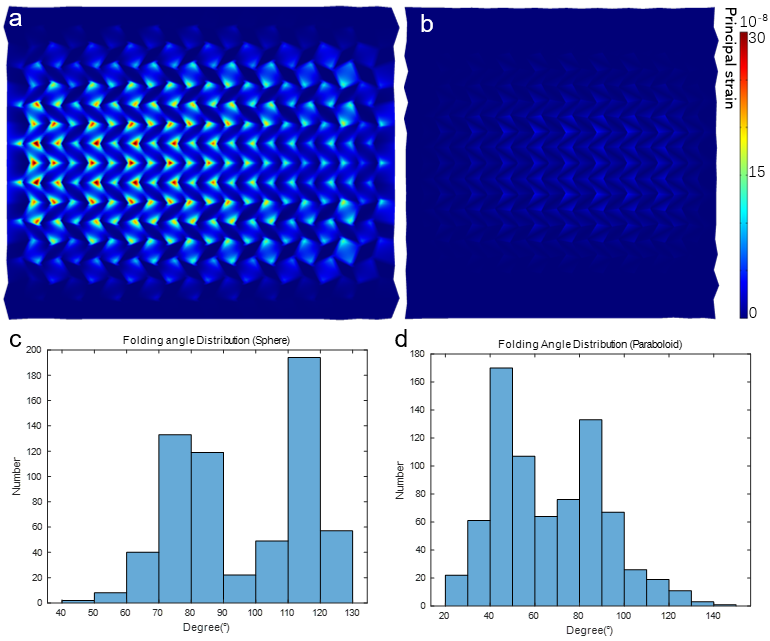


**Fig. S7** **a b** The strain distribution of spherical and saddle origami patterns with idealized creases. **c d** The facets’ folding angles distribution corresponding to **a b**.

The narrow and small facets shorten the distance between LED area and creases, which increase the strain on the facets’ center part. Narrow facets are often accompanied by small angles, which pose a challenge to success. For different target surfaces, the folding angles distribution are shown in Fig. S7 c, d. The angles after folding are mainly distributed between 40° and 120°, which is acceptable when adopting the stiffness modification method that we mentioned in the “Tessellation design and modification” section.

The difference in bending stiffness between facets and creases can significantly reduce the strain on the LED region. Therefore, SU-8 stiffeners were introduced to increase bending stiffness of the facets, and holes were drilled on vertex region to reduce bending stiffness of the creases. According to Li et al ^[31]^, the strain can be reduced for 50% by such methods. Simulation results illustrate that 100μm SU-8 stiffeners reduce the maximum strain to around 1%, which is much less than the strength of the Parylene-C substrate (20%).

**Set up of the optimization problem in COMSOL**

Theoretically, distortion occurs on each parallelogram if the folded Miura-ori structure is forcibly mounted on a curved surface, and strain energy generated accordingly. In our optimization method, we optimize the original origami pattern by entitling the vertexes extra degrees of freedom. Following the “Minimum Total Potential Energy Principle”, vertexes can adjust to the best position for achieving the lowest strain energy of the whole system.

Detailed, the deformation of the original origami pattern to optimized one can be divided into 2 procedures: the initiation process and the optimization process. The initiation process leads the plane sheet to fold as a standard Miura-ori without vertex adjustment (Fig. S8 a). The optimization process forces all the valley vertexes to steadily approach the given curved surface and all the vertexes adjusting themselves to reach the best position (Fig. S8 b).

The purpose of the initiation process is to keep the initial value stable. As Miura-ori is a multi-stable structure, which can be folded into “W-like”, “M-like” or “\v/-like” structures for every 2 adjacent units by alternating the mountain and valley creases. Therefore, a perturbance is introduced to lead the plane sheet into favorable Miura-ori configuration with only one single degree of freedom (DOF). One feasible scheme is to constrain the vertical displacement of all valley vertexes while giving all mountain vertexes upward forces and pushing the structure from one side. In the initiation process, vertexes adjustment $\mathbf{X}\left( \mathbf{X}_{0} \right)$ must be suppressed by introducing a large velocity viscosity to constrain the system properly without additional deformation DOFs.

In the optimization process, the perturbance is released and the viscosity of the vertexes adjustment is lower to allow the optimization to proceed steadily. All the valley vertexes are forced to approach the given curved surface gradually and strain energy $\Pi\left[ \mathbf{x}\left( \mathbf{X} \right) \right]$ is optimized to ensure that bending mainly occurs at the creases throughout the process (Fig. S8 c, d). The relevant schematic diagrams are shown below.


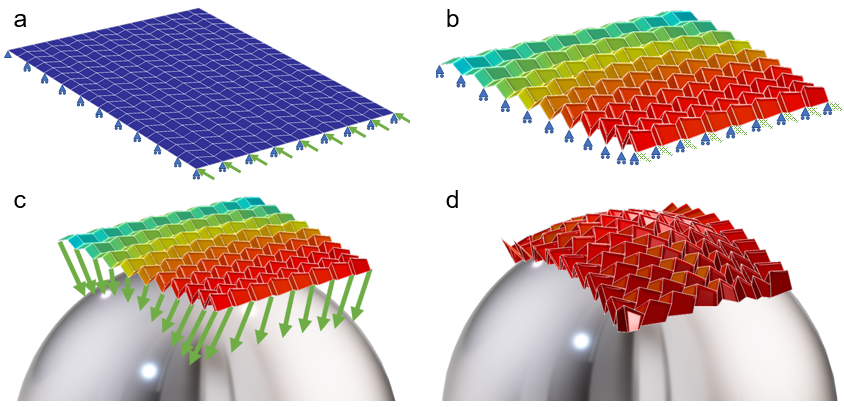


**Fig. S8** Schematic illustrations of the origami optimization method. The initiation process **a b** and the optimization process **c d**.

This optimization is based on the weak form PDE module in COMSOL. The tessellation design of an origami structure is formulated into an optimization problem of the map $\mathbf{X}\left( \mathbf{X}_{0} \right):\mathbf{R}^{2}\to\mathbf{R}^{2}$, which identifies the optimal location $\mathbf{X}$ for each vertex originally located at $\mathbf{X}_{0}$ in a uniform Miura-Ori. The deformation gradient tensor of map $\mathbf{X}\left( \mathbf{X}_{0} \right)$ can be represented as

$$\begin{aligned} \boldsymbol{F}_{Adjust}=\frac{\partial\mathbf{X}}{\partial\mathbf{X}_{\mathbf{0}}},\#\left( 1 \right) \end{aligned}$$

Although the tessellation design only requires the vertex-to-vertex discrete map, we extend the map $\mathbf{X}\left( \mathbf{X}_{0} \right)$ into a continuous map through linear Lagrange interpolation to facilitate numerical calculations.

The deformation of the tessellated origami pattern is described by another map $\mathbf{x}\left( \mathbf{X} \right):\mathbf{R}^{2}\to\mathbf{R}^{3}$, which maps each particle on the 2D plane $\mathbf{X}$ to the location $\mathbf{x}$ in a 3D folded state.

From the perspective of differential geometry, $\boldsymbol{X}$ is a curve coordinate of the folded origami surface $\mathbf{x}$. The tangent vectors on point $(X,Y)$ on the folded surface can be written as

$$\begin{aligned} d\boldsymbol{r}\left( X,Y \right)=\boldsymbol{r}_{X}\left( X,Y \right)dX+\boldsymbol{r}_{Y}\left( X,Y \right)dY\#\left( 2 \right) \end{aligned}$$

where

$$\begin{aligned} \left\{ \begin{aligned} \boldsymbol{r}_{X}\left( X,Y \right)=\left. \frac{\partial\mathbf{x}}{\partial X} \right|_{\left( X,Y \right)}=\left( \frac{\partial x}{\partial X},\frac{\partial y}{\partial X},\frac{\partial z}{\partial X} \right)^{T} \\ \boldsymbol{r}_{Y}\left( X,Y \right)=\left. \frac{\partial\mathbf{x}}{\partial Y} \right|_{\left( X,Y \right)}=\left( \frac{\partial x}{\partial Y},\frac{\partial y}{\partial Y},\frac{\partial z}{\partial Y} \right)^{T} \end{aligned} \right.\#\left( 3 \right) \end{aligned}$$

Therefore, normal vector on point $(X,Y)$ can be calculated as

$$\begin{aligned} \boldsymbol{n}\left( X,Y \right)=\left. \frac{\boldsymbol{r}_{X}\times\boldsymbol{r}_{Y}}{\left| \boldsymbol{r}_{X}\times\boldsymbol{r}_{Y} \right|} \right|_{\left( X,Y \right)}\#\left( 4 \right) \end{aligned}$$

Follow the basic principal in differential geometry, the first and the second fundamental form of the curve surface can be represent as

$$\begin{aligned} I=d\boldsymbol{r}\cdot d\boldsymbol{r}=\left( dX,dY \right) \boldsymbol{C}\left( \begin{matrix} dX \\ dY \end{matrix} \right)\#\left( 5 \right) \end{aligned}$$

$$\begin{aligned} II=-d\boldsymbol{r}\cdot d\boldsymbol{n}=-\left( dX,dY \right)\boldsymbol{K}\left( \begin{matrix} dX \\ dY \end{matrix} \right)\#\left( 6 \right) \end{aligned}$$

where $\boldsymbol{C}$ and $\boldsymbol{K}$ are also known as metric tensor and Riemann curvature tensor respectively. Gaussian curvature can also be represented as $\kappa_{G}=\frac{\det\boldsymbol{K}}{\sqrt{\det\boldsymbol{C}}}.$

To realize such algorithm in COMSOL, we take $\mathbf{X}_{\mathbf{0}}$ as the unique reference state coordinate. Map $\mathbf{x}\left( \mathbf{X}_{0} \right)$ transform the flat Miura-origami into the final tessellation design. Therefore, the deformation gradient of $\mathbf{x}\left( \mathbf{X} \right)$can be represented as

$$\begin{aligned} \frac{\partial\mathbf{x}}{\partial\mathbf{X}}=\frac{\partial\mathbf{x}}{\partial\mathbf{X}_{\mathbf{0}}}\frac{\partial\mathbf{X}_{\mathbf{0}}}{\partial\mathbf{X}}=\frac{\partial\mathbf{x}}{\partial\mathbf{X}_{\mathbf{0}}}{\boldsymbol{F}_{\mathrm{Adjust}}}^{-\mathbf{1}}\#\left( 7 \right) \end{aligned}$$

With the contributions from both out-of-plane bending and in-plane stretching are taken into account, the total elastic energy takes the form

$$\begin{aligned} \Pi\left[ \mathbf{x}\left( \mathbf{X} \right) \right]=\int_{A} \left[ \xi\left( C_{\alpha\alpha}+\frac{1}{\det\mathbf{C}}-3 \right)+K_{\alpha\beta}K_{\alpha\beta} \right]dA,\#\left( 8 \right) \end{aligned}$$

where repeated indices in Greek imply a summation over the in-plane dimensions.

According to the numerical integration method bulit-in in COMSOL, the additional manufacture and applications constraints can also be achieved easily. If we reduce the number of Gaussian quadrature points to 1 for each element, we are evaluating the center value for the integrand and multiply it to the element area. It is worth mentioning that the first order Gaussian quadrature rule can guarantee a first-order accuracy. Therefore, the additional constraints can be written as

$$\begin{aligned} \eta_{1}\sum_{e} \mathrm{abs}\left( \bar{x}_{\xi}-\bar{x}_{\xi}^{0} \right)=\frac{\eta_{1}}{A}\int_{A} \left[ \mathrm{abs}\left( x-x_{0} \right) \right]dA,\#\left( 9 \right) \end{aligned}$$

$$\eta_{2}\sum_{e} \left[ \int_{e} \det\left( \boldsymbol{F}_{Adjust} \right)dA_{e}-A_{e} \right]^{2}=\frac{\eta_{2}}{A}\int_{A} \left[ \det\left( \boldsymbol{F}_{Adjust} \right)-1 \right]^{2}dA, \left( 10 \right)$$

where $\mathrm{abs}\left( \bar{x}_{\xi}-\bar{x}_{\xi}^{0} \right)$ is a piecewise linear function and $\det\frac{\partial\mathbf{X}}{\partial\mathbf{X}_{0}}$ is a piecewise constant value.

To enhance the convergence speed and stability of the optimization problem, we can also add viscous term as

$$\begin{aligned} \zeta_{vis}\cdot\int_{A} \left( X-X_{0} \right)\frac{dX}{dt}dA\#\left( 11 \right) \end{aligned}$$

where $\zeta_{vis}$ is a positive damping parameter.

To skip the initial singular case, we also need to imply a initial perturbation to guide it deform into a folded Miura origami structure first and exert the strain energy after that.

A suggested set of weight coefficients is list as: $\xi=0.0004, \eta_{1}=0.5\cdot A, \eta_{2}=1\cdot A,\zeta_{vis}=0.0001$.
